# The impact of health symptoms on health-related quality of life in early-stage breast cancer survivors

**DOI:** 10.1007/s10549-019-05433-3

**Published:** 2019-09-11

**Authors:** K. M. de Ligt, M. Heins, J. Verloop, N. P. M. Ezendam, C. H. Smorenburg, J. C. Korevaar, S. Siesling

**Affiliations:** 1 Department of Research and Development, Netherlands Comprehensive Cancer Organisation, PO Box 19079, 3501 DB Utrecht, The Netherlands; 2 Department of Health Technology and Services Research, Technical Medical Centre, University of Twente, Enschede, The Netherlands; 3 NIVEL Netherlands Institute for Health Services Research, Utrecht, The Netherlands; 4 Department of Medical and Clinical Psychology, Tilburg University, Tilburg, The Netherlands; 5 Department of Medical Oncology, Netherlands Cancer Institute - Antoni van Leeuwenhoek, Amsterdam, The Netherlands

**Keywords:** Breast neoplasms, Aftercare, Survivorship, Health-related quality of life, Late effects

## Abstract

**Purpose:**

In breast cancer patients, treatment-related health symptoms can occur that may affect their health-related quality of life (HRQoL). This study aimed to determine the impact of health symptoms on HRQoL in breast cancer patients up to 5 years after diagnosis.

**Methods:**

Females surgically treated for early-stage breast cancer diagnosed between 2012 and 2016 (*n* = 876) were selected from the Netherlands Cancer Registry and invited for a survey about current health symptoms (‘Symptoms and Perceptions questionnaire’, SaP) and HRQoL (‘EORTC-QLQ-C30’). From the latter, functioning and global health were included. Mean scores were compared to norm population scores (*T* test). Multivariable linear regression analyses were performed to determine the association between health symptoms and global health and functioning.

**Results:**

404 patients (46%) responded. The median age was 62.2 ± 10.9 years. Respondents had significantly lower mean scores for role, cognitive, emotional, and social functioning than the general population. The most frequently reported health symptoms were musculoskeletal (including pain/complaints in lower/upper extremities/back/neck; 71%) and central nervous system symptoms (including concentration impairment, dizziness, neuralgia; 66%), and fatigue (63%). While most symptoms affected functioning, irrespective of time since diagnosis, especially fatigue, musculoskeletal, central nervous system, and gastrointestinal symptoms were significantly associated (*p* < 0.05) with lower functioning.

**Conclusions:**

The majority of health symptoms that occur after breast cancer treatment were associated with lower functioning of patients in daily life. This paper urges healthcare providers to support breast cancer patients in alleviating or coping with health symptoms, even years after end of treatment, to improve their functioning.

**Electronic supplementary material:**

The online version of this article (10.1007/s10549-019-05433-3) contains supplementary material, which is available to authorized users.

## Introduction

For early-stage breast cancer, five-year survival rates are relatively high and have been increasing over the recent years [1, 2], with current rates in Europe and North America exceeding 85% [1, 3]. This is mainly due to early detection by improved screening [2, 4] and improvements in multidisciplinary treatment [4, 5]. Although breast cancer survivors report a relatively high health-related quality of life (HRQoL) [6–9], effects of disease and treatment may lower HRQoL. These effects can impact all aspects of life, including physical, emotional, psychosocial, and cognitive well-being [10–12], and include lymphedema, pain, and movement restrictions in the arm and shoulder [10, 12–14], premature menopause, neuropathy [11–15], bone loss [10, 13, 14], cardiotoxic effects [10, 13, 14], fatigue, insomnia, depression, cognitive dysfunction [10–12, 14, 15], and sexual problems [10, 11, 14]. In general, a higher symptom burden was associated with lower HRQoL [16, 17].

Clinical guidelines recommend that patients receive at least 5 years of follow-up care to detect recurrent disease and to manage physical and psychosocial sequelae [18–20]. Even though survivorship care has become an increasingly important part of care, there are concerns that benefits in treatment of breast cancer do not lead to similar benefits in psychosocial, functional, and sexual well-being [21]. Both detection and management of late and side effects of breast cancer and its treatment are widely addressed as a research priority for the recent future [22, 23]. Patient-Reported Outcome Measures (PROMs) are suggested as symptom detection method [21, 23], but implementation in daily practice is hampered [21, 24, 25]. Furthermore, support in coping with symptoms may be insufficient, as patient-reported health symptoms consistently over time (years) after diagnosis [9, 11, 26, 27]. Wu et al. describe that 92% of patient-reported residual symptoms 1 year after diagnosis, and 61% reporting pain, fatigue, and sleep disturbance up to 5 years after diagnosis [27]. Large unmet needs were found for information, detection, and management of physical impairments [9, 12], cognitive impairments [9], sexual functioning and enjoyment [9, 28], menopausal disorders [9], and anti-hormonal treatment effects as hot flashes [9, 29, 30]. These unmet needs often mediate a lower HRQoL, and were associated with worse perceived physical and mental health [27, 31]. In general, residual treatment-related health symptoms were associated with lower QoL [27, 31], disability, and increased healthcare use [27].

To improve the follow-up care, knowledge about all potential short and long-term treatment-related health symptoms and their impact on HRQoL is needed [23]. Specifically, it may be most effective to detect and, if possible, successfully unburden the health symptoms that are significantly associated with a lower HRQoL. The effects of symptoms on HRQoL up to 1 year after treatment are commonly known [11, 17], and for the long term, these effects were explored for frequently prevalent health symptoms such as fatigue, sleep, depression, and pain [9, 27]. Still, however, for the complete range that could occur in breast cancer patients [10, 13–15, 17, 26], the association of health symptoms with long-term HRQoL was not explored [17, 27, 32]. This is a potentially important input for follow-up tools and guidance, which was addressed as a research need for breast cancer by the ESMO expert panel [23].

HRQoL is a multi-domain construct, typically including (overall) health perceptions, functioning, and symptoms. In this study we aimed to determine the impact of prevalent health symptoms on health perception and functioning in breast cancer patients up to 5 years after diagnosis.

## Methods

This cross-sectional survey study utilized the data collected in our previous study [26]. Surgically treated female breast cancer patients (> 18 years), diagnosed with early-stage disease (stage I–III) between 2012 and 2016 were selected from the Netherlands Cancer Registry (NCR), a national database that has documented population-based data about cancer incidence, diagnosis, and treatment [33]. For each participating hospital (*N* = 20), fifty patients were randomly selected (*N* = 1000). In deliberation with these hospitals, patients who did not receive active follow-up, who were currently receiving treatment for secondary or recurrent disease, who could not read or write Dutch, or had no recent contact information, were excluded (*n* = 124). Patients (*n* = 876) were then invited by the hospital administrations to complete the survey through the online PROFILES (‘Patient-Reported Outcomes Following Initial treatment and Long-term Evaluation of Survivorship’) Registry survey application [34]. Invitations were sent between September 2017 and March 2018, responses were collected until May 2018. Participants gave consent for processing their coded responses and merging these with their clinical data available in the NCR. The use of NCR data in this study was approved by the NCR Privacy Review Board. Formal approval from an ethics committee was not required as the Dutch Medical Research (Human Subjects) Act did not apply for this study.

The survey (Appendix 1) consisted of three existing questionnaires and several self-composed questions: (1) HRQoL over the past 4 weeks; (2) health symptoms and diseases over the past year; (3) sociodemographic characteristic (age, highest completed level of education) and disease status (current treatment status, presence of comorbid disease at time of survey). HRQoL was measured through the EORTC-QLQ-C30 Quality of Life Questionnaire for Cancer. The QLQ-C30 includes a 2-item global health status/QoL scale, five multi-item functional scales (physical, role, emotional, cognitive, social functioning), and nine symptom scales or items. Answer scales ranged from ‘not at all’ to ‘very much’ in four steps. After transformation, scores ranged from 0 to 100, with high scores depicting good global health and functioning [35]. Forty-two health symptoms were presented (‘health problem present: yes/no’) in the validated Symptoms and Perceptions questionnaire [36], supplemented with breast cancer-specific health symptoms from the literature [10, 12–14]. Health symptoms were categorized in ten categories based on organ system. Comorbidities at time of survey were based on Sangha et al. [37], comprising the following diseases (as diagnosed by a physician) rather than separate health symptoms: other types of cancer, pulmonary, cardiovascular, gastrointestinal, urogenital, musculoskeletal, neurological, metabolic/coagulation, or infectious diseases.

A normative population sample (*n* = 1.105 women, surveyed in 2013) was retrieved from CentERdata [38]. Breast cancer patients were not excluded from this population cohort; we did look into the results for the population cohort without cancer patients as sensitivity analysis.

### Analyses

First, the respondent characteristics (age, year of diagnosis, type of surgery, stage of disease, and type of hospital) were compared to non-respondent characteristics to assess generalizability (*χ*^2^, level of significance *p* < 0.05). Respondent characteristics, health symptoms, and global health and functioning were reported. Second, multivariable linear regression analyses were performed to determine the effect of health symptoms on global health and functioning. Variables were selected based on significance; levels of significance of 0.10 and 0.05 were applicable for univariable and multivariable analyses, respectively. Backward selection was applied to reach parsimonious models. We corrected for time since diagnosis [6, 39], age [6, 27, 39], presence of comorbid disease(s) [6, 27], and the level of education [6, 27]; these variables were selected based on the literature and availability in our dataset. We also corrected for breast reconstruction, as we expected that this treatment modality had an independent and positive effect on HRQoL [40, 41]. We did not correct for other treatment modalities.

HRQoL scores for the respondents and norm population were compared through *T* testing. Cohorts were matched 1:1 based on age (categories: < 50, 50–59, 60–69, 70+). Furthermore, scores were stratified by time since diagnosis (> 2, 2–4, 4+ years) and tested through one-way ANOVA. For both, a level of significance of *p* < 0.05 was practised.

All analyses were performed in STATA SE14.2 [42].

## Results

Completed surveys were received from 46% of the invited patients (404/876). Respondents and non-respondents did not differ significantly based on patient and treatment characteristics, although respondents were slightly underrepresented in the youngest and oldest age category (< 50 years, 20% vs. 25%; 70+ years, 17% vs. 23%; *p* = 0.010, Supplementary Table 1).

Table 1 reports the respondents’ patient, tumour, and treatment characteristics. Mean age was 62.0 ± 10.9 years, and one or more comorbidity was present in 48% of patients at the time of survey. Patients had been treated with either breast conserving surgery (59%) or mastectomy (41%). Additionally, patients had received treatment with radiotherapy (72%), chemotherapy (49%), and anti-hormonal therapy (57%). Table 2 presents patient-reported health symptoms categorized by the organ system. The most commonly reported were health symptoms of the musculoskeletal system (71%) and central nervous system (66%), and fatigue (63%).

**Table 1:** Respondent characteristics (*n* = 404)

	*N* (404)	%
**Patient characteristics**		
Age (in years) at time of survey		
Mean (SD, range)	62.20 (11.0, 27.5–91.6)	
< 50	57	14
50–59	109	27
60–69	136	33
70+	102	25
Time (in years) between diagnosis and survey		
< 2	83	21
2–4	177	44
> 4	144	36
Highest completed level of education^a,b^		
Secondary education or lower	122	30
Medium vocational training	170	42
High vocational training	108	27
Number of comorbidities^a,b^		
0	188	47
1	131	33
2≥	61	15
Unknown	24	6
**Tumour characteristics**		
Year of diagnosis		
2012	54	13
2013	92	23
2014	86	21
2015	89	22
2016	83	21
Stage		
I	186	46
II	174	43
III	44	11
Hormone-receptor status^a^		
HR-positive	287	71
HR-mixed	53	13
HR-negative	62	15
Tumour grade^a^		
1	95	24
2	176	44
3	95	24
**Treatment characteristics**		
Treatment status at time of survey		
Completed	180	45
Currently receiving anti-hormonal therapy	173	43
Currently receiving other treatment	29	7
Surgery		
Breast conserving surgery	238	59
Mastectomy	160	40
Axillary dissection	85	21
Immediate breast reconstruction	36	9
Adjuvant treatment		
Radiotherapy	291	72
Chemotherapy	196	49
With trastuzumab	50	12
Anti-hormonal therapy	232	57
**Hospital characteristics**		
Hospital type^c^		
General hospital	166	41
Teaching/academic hospital	238	59
Hospital volume^d^		
Low	157	39
Medium	88	22
High	159	39

^a^Totals do not add up due to missing values

^b^Patient-reported

^c^Hospitals were categorized as general, teaching, or academic hospitals

^d^Number of surgically treated non-metastatic breast cancer patients per year (average over 2012–2016), categorized as low (< 100), medium (100–149), and high (> 150) volume

**Table 2:** Categories of patient-reported symptoms

	*N* (404)	%
Fatigue	256	63
Cardiac: palpitations, chest pain or tightness	77	19
Respiratory: cough, complaints in the nose, shortness of breath	124	31
Gastrointestinal: dry mouth, diarrhoea/constipation, gastric or abdominal complaints, nausea	160	40
Urinary complaints: difficulties with urinating in general	34	8
Central nervous system: memory/concentration, tingling hands/feet (neuralgia), irritation of eyes, dizziness/vertigo, headache, earache or ear complaints, hypersensitivity to light or sound	267	66
Skin: hair loss, skin problems	153	38
Psychological: insomnia, agitation/irritability, anxiety, depressive feelings, sudden feelings of stress or crisis, increased in use of drugs or alcohol	214	53
Reproductive system: menopausal complaints, weight increase/decrease, problems with sex or sexuality, infertility	220	54
Breast: hypersensitivity in breast area, pain/swelling scars in breast area, axillary complaints (incl. lymphoedema), skin problems in breast area	218	54
Musculoskeletal: pain/complaints in upper extremities, pain/complaints in lower extremities, neck or shoulder pain/complaints, myalgia/muscle strain, back pain/complaints, movement restrictions in arm, fractures	285	71
Reported in De Ligt et al. [26]		

### HRQoL compared to norm population

**Fig. 1:**
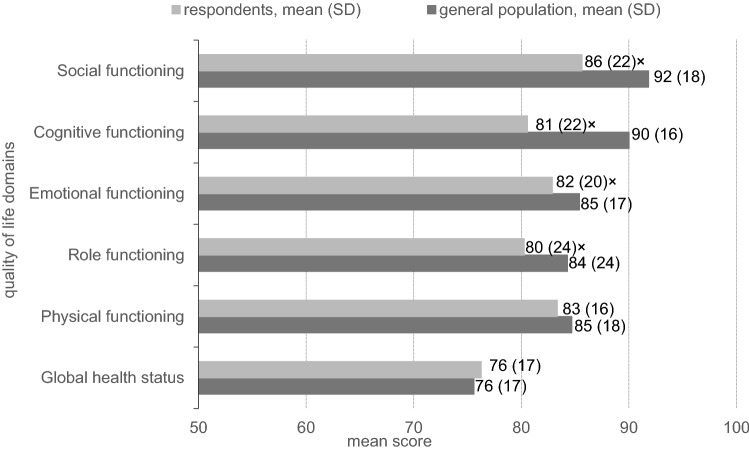
Mean HRQoL, compared to the general population. Scales range from 0 to 100, with high scores depicting good global health and functioning × significant difference (*p* < 0.05, *T* test) respondents vs general population

Figure 1 reports the mean global health and functioning for the respondents and reference population. The mean global health score for respondents (76.3 ± 17.2) did not differ significantly from the general population score (75.6 ± 16.9). Although mean scores for all individual functioning domains were 80 or higher, these scores were significantly lower than those in the general population for role (80.3 ± 23.6 vs. 84.3 ± 23.6; *p* = 0.016), emotional (82.9 ± 19.9 vs. 85.4 ± 16.9; *p* = 0.053), cognitive (80.6 ± :21.9 vs. 90.0 ± 15.9; *p* < 0.001), and social (85.6 ± 21.6 vs. 91.8 ± 17.7; *p* < 0.001) functioning. Excluding cancer patients from the general population sample did not alter our findings.

Specifically in younger patients (age < 50 years), physical functioning was significantly better (5 points difference), while cognitive (11 points) and social (9 points) were significantly worse than in older patients (results not shown). When stratified by years since diagnosis, the mean HRQoL scores did not statistically differ over time (Supplementary Table 2).

When stratified by the number of symptom categories, mean scores for all domains were significantly lower when more symptoms were reported (Supplementary Table 3). Only 12 respondents (3%) reported zero symptoms in either of the eleven categories of symptoms, while 37%, 50%, and 11% reported 1–4, 5–8, and 8–11 categories of symptoms, respectively. Mean global health scores ranged between 91.7 and 64.9 when zero and 8–11 categories of health symptoms were reported (*p* < 0.001), respectively. For the functioning scales, mean scores ranged between 100 (social functioning) and 63.1 (cognitive functioning) for zero and 8–11 categories of health symptoms reported, respectively. Frequently reported dyads of health symptoms were reported in Supplementary Table 4. Especially fatigue, symptoms of the central nervous system, and musculoskeletal symptoms were reported frequently in combination with other symptoms.

### The effect of health symptoms on HRQoL scores

Table 3 presents the associations between prevalent health symptoms and functioning, adjusted for covariates and after backward selection. A *β* of -x indicated a negative association between the health problem and global health or functioning of x points. All included domains were significantly negatively associated with either one of the categories of health symptoms. Cognitive functioning was affected by a range of health symptoms, including cardiac (*β*: − 4.4, *p* = 0.002), gastrointestinal (*β*: − 4.3, *p* = 0.042), renal and urinary (*β*: − 9.7, *p* = 0.006), central nervous system (*β*: − 16.2, *p* < 0.001), and psychological (*β*: − 5.2, *p* = 0.017) health symptoms. Furthermore, health symptoms burdened different aspects of quality of life. For instance, musculoskeletal health symptoms affected global health status (*β*: − 4.2, *p* = <0.028), and physical (*β*: − 7.6, *p* = <0.001), role (*β*: − 9.5, *p* < 0.001), emotional (*β*: − 5.0, *p* = 0.019), and social (*β*: − 6.3, *p* = 0.016) functioning. Last, associations were of different magnitudes. The largest effects were found for the associations between health symptoms of the central nervous system and cognitive functioning (*β*: − 16.2, *p* < 0.001), and fatigue and role functioning (*β*: − 14.1, *p* = <0.001).

**Table 3:**
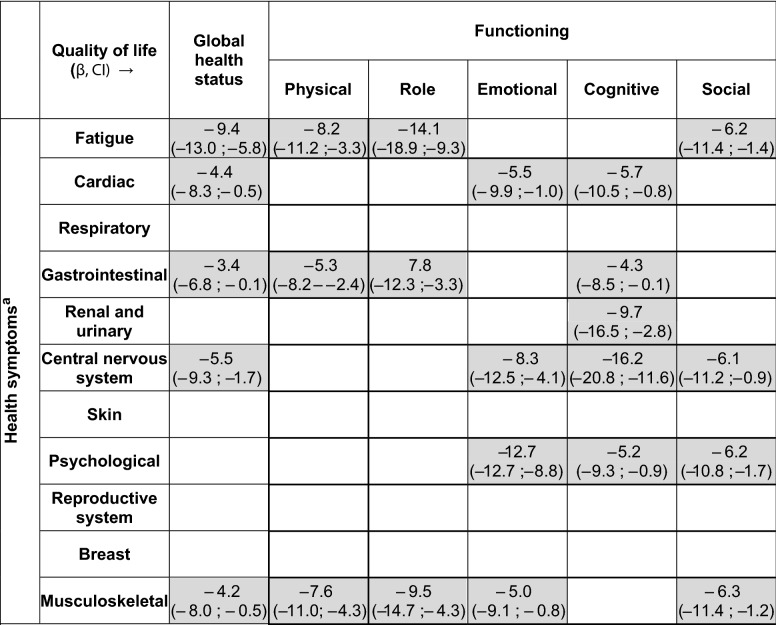
Association between health symptoms and HRQoL functioning domains through multivariable linear regression

Cells are empty when factors were not significant in univariate testing and thus not included in multivariate testing, or were excluded through backward selection in the multivariate analyses. Analyses were corrected for age at time of survey, presence of comorbid diseases, highest completed level of education at time of diagnosis, and breast reconstruction

*CI* confidence interval, *β* association of x points of health problem on HRQoL

^a^In categories, reference categories for health symptoms were patients who not reported health symptoms in this category

Supplementary Table 5 includes all covariates included in the multivariate analyses. Time since diagnosis was not significantly associated with functioning. Higher age was associated with significantly lower physical functioning (60–69: *β*: − 6.8, *p* = 0.002; 70+ : *β*: − 13.5, *p* < 0.001) and better cognitive functioning (60–69: *β*: 7.4, *p* = 0.014; 70+ : *β*: − 7.8, *p* = 0.018). Presence of comorbid disease was negatively associated with global health (*β*: − 5.5, *p* = 0.001), physical functioning (*β*: − 6.1, *p* < 0.001), and role functioning (*β*: − 8.5, *p* < 0.001). Immediate breast reconstruction was associated with a higher global health (*β*: 7.7, *p* = 0.005) and emotional functioning (*β*: 8.4, *p* = 0.007).

## Discussion

Mean global health in breast cancer patients up to 5 years after diagnosis was comparable to that in the general population (76.3 vs. 75.6). Mean scores of 80 or higher were found for functioning, although these were significantly lower than those in the general population for role, emotional, cognitive, and social functioning. Almost all the reported health symptoms were significantly negatively associated with either one of the functioning scales, but were most pronounced for musculoskeletal health symptoms, fatigue, health symptoms in the central nervous system, and gastrointestinal health symptoms.

We found that HRQoL in breast cancer survivors up to 5 years after diagnosis is relatively high compared to the general population, alike previous literature [6, 7]. In line with the previous EORTC-QLQ-C30 measurements, standard deviations were quite wide [17, 32, 43–45]. We believe that especially the domains for which a lower mean score lower than those in the general population was reported are important to breast cancer patients, although not all reported differences may be regarded clinically relevant [46, 47]. For cognitive functioning, we found a 9-point difference; a mean difference of 9–14 points was regarded a medium effect, suggesting the difference we found is clinically relevant. For social functioning, we found a 6-point difference, which is regarded a small effect (5–11 points mean difference). For the other domains, differences found were of trivial effect and thus of less clinical relevance. However, as an individual may encounter effects simultaneously on multiple domains, we suggest trivial or small differences should not be immediately neglected. Our results describe that global health and functioning is significantly lower by a higher symptom prevalence. Especially fatigue, and central nervous system and musculoskeletal symptoms were reported frequently in combination with other symptoms. Thus, several small mean differences may add up to large and multifaceted effects on global health and functioning. We believe this deserves more awareness in clinical practice.

In line with the literature [44], we found no statistically significant difference between the respondents and the general population for global health and physical functioning. Although seemingly contradictory to the lowered functioning scores we found, similar results were reported in other studies [27]. A possible explanation may be a re-evaluation of general health perception in breast cancer patients, or so-called ‘response shift’ [48]. However, it may be as well that the global health domain is a less discriminating measurement domain than other domains. Blome et al. [48] describe that it is assumed that HRQoL is a more or less universal concept, while in fact, a certain objective impairment does not necessarily lead to the same reduction of HRQoL in any patient.

Negative associations with functioning were especially found for fatigue and musculoskeletal, central nervous system, and gastrointestinal health symptoms. These first two were found by Arndt et al. [32] as well in patients up to 1 year after diagnosis, while after a year other symptoms than acute symptoms became relevant [11, 32]. Furthermore, we found that the mean scores did not differ according to time since diagnosis. Literature found reduced HRQoL up to 5 years after diagnosis [43, 44], but more improvements onwards as well [39], underlining the importance of longitudinal data to determine time effects for HRQoL.

### Study limitations

Several forms of bias may apply to patient-reported data, including recall bias, report bias, selection bias, and survivorship bias. As a result of recall bias, the reported symptoms may have been underrepresented when patients were not able to recall all symptoms they encountered in the previous year. Although recall bias was not applicable for the assessment of HRQoL at time of survey, response shift may lead to different interpretations of HRQoL over time [48]. Also, symptoms may have been either overestimated if only patients with many health symptoms participated, or underestimated by reluctance towards reporting for instance sexuality problems [38]. Furthermore, we excluded patients who could not read or write Dutch, thereby risking to exclude a vulnerable part of the patient population: non-responding patients in observational patient-reported studies have different sociodemographic and clinical characteristics, and may have systemically lower HRQoL scores [49]. As patients with less favourable sociodemographic characteristics may have been exposed to other risk factors affecting both breast cancer risk and HRQoL, information about healthy behaviour would be beneficial in further interpreting the results. As a result of survivorship bias, patients with relatively favourable disease characteristics may have been included.

### Implications for practice

We were able to confirm that a higher symptom burden was associated with a lower HRQoL [16, 17]. Therefore, it may be most effective to detect and manage or unburden the treatment-related health symptoms that were significantly associated with a lower HRQoL, and thus provide follow-up care that is valuable to the daily functioning of breast cancer patients. For instance, guidelines provides recommendations for physical therapy for the effects of musculoskeletal health symptoms as arm/shoulder function and lymphoedema [18, 19]. However, such clear recommendations are not available for all reported negative associations. Fatigue was prevalent in 9–100% of cancer survivors [9, 15, 17, 26, 27, 44] and has a large impact on HRQoL and functioning [9, 15, 17, 27, 32]. Exercise, cognitive-behavioural therapy, and education about coping were found to be effective against cancer-related fatigue [10, 50, 51], however, fatigue was found to be a very persistent problem, lasting up to 10 years in one-fourth to one-third of breast cancer patients [15]. Moreover, of the 63% of respondents who reported fatigue, only a third reported this to her physician [26], suggesting underdetection. Furthermore, a large negative association was found between health symptoms of the central nervous system and cognitive functioning, probably caused by the large proportion of patients (42.6%) reporting memory and concentration symptoms. Even though cognitive dysfunction is frequently reported among (breast) cancer patients, not much is known about its aetiology, and proven effective interventions are lacking [10, 15, 52]. Last, we found negative associations between gastrointestinal problems and global health, and physical, role, and cognitive functioning. In literature, gastrointestinal symptoms as diarrhoea, nausea, appetite loss, and constipation were reported less frequently over time, by 15% to 20% of patients [44], which we could confirm [26]. Gastric or abdominal complaints were reported in 13% of patients. However, over 50% of them reported healthcare use for this symptom, which was nearly the highest reported use of care for the range of included symptoms [26]. Literature reported that the majority of gastro-complaints declined over time [11, 44], still, the high use of care could indicate many patients struggle with these symptoms long after diagnosis.

These examples illustrate not only the importance of detection and acknowledgement of health symptoms, but also the importance of research towards successful detection and intervention for these symptoms [53], so that guidelines can provide guidance in dealing with them. Survivorship research is a priority research area for breast cancer, including follow-up tools to assess quality of life in long-term survivors [23].

We measured symptoms through the SaP questionnaire [36], rather than through the EORTC-QLQ-C30 symptom domains. The SaP assesses a broader range of non-specific symptoms, aiming to include symptoms from all organ systems. We hypothesized that a broader selection of symptoms than currently included in the QLQ-C30 is of relevance for longer-term survivors [27]. Our results confirm this hypothesis. For instance, menopausal (40%) and memory and concentration complaints (43%) were commonly reported, but are not or limitedly included in the QLQ-C30 and BR-23 breast cancer module. This hypothesis was confirmed by Van Leeuwen et al. [11]. We endorse Van Leeuwen et al. that development of a survey specifically for cancer survivors would address a gap in assessing issues of a more chronic nature [11].

## Conclusion

Early-stage breast cancer patients up to 5 years after diagnosis reported significantly lower mean scores than the general population for all functioning domains but physical functioning, and scores did not differ statistically by time since diagnosis. The majority of symptoms prevalent after breast cancer treatment was associated with lower functioning of patients in daily life. This urges healthcare providers to support patients in alleviating or coping with treatment-related health symptoms, even years after end of treatment, to improve functioning of breast cancer survivors.

## Electronic supplementary material

Below is the link to the electronic supplementary material.

Supplementary material 1 (DOCX 55 kb)
