# Supplementary material for: The impact of health symptoms on health-related quality of life in early-stage breast cancer survivors
Source: Breast Cancer Res Treat. 2019 Sep 11;178(3):703–11. doi: 10.1007/s10549-019-05433-3 (PMC6817812; doi:10.1007/s10549-019-05433-3)
Supplement: Supplementary file 1 — Supplementary material 1 (DOCX 55 kb) [file 10549_2019_5433_MOESM1_ESM.docx]

**Appendix 1**

| **0.** | **What is your date of birth?** |
| --- | --- |
|  | ..-..-19.. |

| **1.** | **In general, would you say your health is:** |
| --- | --- |
|  | 1 - Excellent  2 - Very good  3 - Good  4 - Fair  5 - Poor |

| **2.** | **I seem to get sick a little easier than other people** |
| --- | --- |
|  | Definitely true – Mostly true - Don't know – Mostly false – Definitely false |

| **3.** | **I am as healthy as anybody I know** |
| --- | --- |
|  | Definitely true – Mostly true - Don't know – Mostly false – Definitely false |

| **4.** | **I expect my health to get worse** |
| --- | --- |
|  | Definitely true – Mostly true - Don't know – Mostly false – Definitely false |

| **5.** | **My health is excellent** |
| --- | --- |
|  | Definitely true – Mostly true - Don't know – Mostly false – Definitely false |

| **6.** | **Compared to one year ago, how would you rate your health in general now?** |
| --- | --- |
|  | 1 - Much better now than one year ago  2 - Somewhat better now than one year ago  3 - About the same  4 - Somewhat worse now than one year ago  5 - Much worse now than one year ago |

| **7.** | **Do you currently suffer from one or more of undermentioned diseases?** |
| --- | --- |
|  | Any other type of cancer – lung disease - cardiovascular disease – gastro-intestinal disease – illness of urinary or reproductive system – musco-skeletal disease - central nerve system – illness of metabolism or coagulopathy - infectious disease – none - other |
|  | **In case you do: for which of these did you receive treatment?** |

| **8.** | **Which of the following health problems did you experience over the past years? And for which of these health problems did you visit a (primary care) physician? *** |
| --- | --- |
|  | Palpitations  Chest pain/pressure  Shortness of breath  complaints nose  Cough  Stomach/abdomen complaints  Dry mouth  Nausea  Diarrhea/constipation  Urinary complaints  Memory/concentration  Irritation to the eye(s)  Dizzyness  Head ache  Ear pain or complaints  Tingling hands/feet  Hypersensitive for light  Skin problems  Hair loss  Anxiety  Depressive feelings  Sudden feelings of stress or crisis  agitation/irritability  Insomnia  Increase drugs/alcohol use  Problems sex(uality)  Menopausal complaints  Weight increase/decrease  Infertility  Pain/swelling scars  Hypersensitive breast area  Complaints axilla (incl. lymph edema)  Skin problems breast area  Neck or shoulder pain/complaints  Back pain/complaints  Pain/complaints upper extremities  Fractures  Pain/complaints lower extremities  Myalgia/muscle strain  Movement restrictions arm |

| **9.** | **What was your most important health problem reported in the list above? Fill in only one health problem.** |
| --- | --- |
|  |  |

**[EORTC QLQ-C30 Questionnaire]**

| **13.** | **What is your highest completed education? (completed with diploma or certificate)** |
| --- | --- |
|  | No education – lower education – middle education – higher education - other |

| **14.** | **Do you have a partner?** |
| --- | --- |
|  | Yes - No |

| **15.** | **Do you have children?** |
| --- | --- |
|  | Yes, they live with me – Yes, they live on their own - No |

| **16.** | **What description is most applicable to you at this moment? (please tick one answer)** |
| --- | --- |
|  | Attending school/education – paid employment – unemployed/seeking work – incapacitated – housewife - retirement |

| **17.** | **Are you currently under treatment for your breast cancer?** |
| --- | --- |
|  | No, I am finished with my treatment – Yes, I currently receive anti-hormonal treatment – Yes, I receive [fill in which treatment you currently receive] |

* Symptoms and Perceptions questionnaire

**Supplementary Table 1: characteristics respondents (n=408) vs non respondents (n=592)**

| **Patient and tumour characteristics** | | **Responders** | | **Non-responders** | | **P^a^** |
| --- | --- | --- | --- | --- | --- | --- |
|  |  | **N = 408** | **%** | **N =592** | **%** |  |
| Age | <50 | 82 | 20% | 147 | 25% | 0.010 |
|  | 50-59 | 127 | 31% | 157 | 27% |  |
|  | 60-69 | 128 | 31% | 150 | 25% |  |
|  | 70+ | 71 | 17% | 138 | 23% |  |
| Year of diagnosis | 2012 | 55 | 13% | 116 | 20% | 0.120 |
|  | 2013 | 93 | 23% | 115 | 19% |  |
|  | 2014 | 86 | 21% | 116 | 20% |  |
|  | 2015 | 89 | 22% | 118 | 20% |  |
|  | 2016 | 85 | 21% | 127 | 21% |  |
| Type of surgery* | Breast conserving | 162 | 40% | 223 | 38% | 0.769 |
|  | Amputation | 240 | 59% | 360 | 61% |  |
| Stage | 1A | 175 | 43% | 265 | 45% | 0.205 |
|  | 1B | 13 | 3% | 24 | 4% |  |
|  | 2A | 112 | 27% | 173 | 29% |  |
|  | 2B | 64 | 16% | 59 | 10% |  |
|  | 3A | 24 | 6% | 41 | 7% |  |
|  | 3B | 9 | 2% | 10 | 2% |  |
|  | 3C | 11 | 3% | 20 | 3% |  |
| Type of hospital^b^ | General hospital | 178 | 44% | 274 | 46% | 0.277 |
|  | Teaching (topclinical) hospital | 221 | 54% | 297 | 50% |  |
|  | Academic hospital | 9 | 2% | 21 | 4% |  |
| a Chi-square tested.  b Hospitals were categorised as either general, teaching, or academic hospitals.  *do not add up due to missing values | | | | | | |

**Supplementary Table 2: Mean quality of life domain scores per year since diagnosis**

|  | **Time since diagnosis** | | | | | | |
| --- | --- | --- | --- | --- | --- | --- | --- |
| **HRQoL**  **domains** | **<2** |  | **2-4** |  | **4+** |  |  |
|  | **mean** | **SD** | **mean** | **SD** | **mean** | **SD** | **p** |
| **Global health status** | 77.9 | 16.6 | 75.7 | 27.1 | 76.0 | 8.3 | 0.616 |
| **Physical functioning** | 84.0 | 14.9 | 83.9 | 16.2 | 82.3 | 17.5 | 0.641 |
| **Role functioning** | 81.1 | 24.5 | 79.3 | 22.4 | 81.0 | 24.4 | 0.777 |
| **Emotional functioning** | 83.3 | 16.9 | 83.1 | 19.5 | 82.4 | 22.1 | 0.927 |
| **Cognitive functioning** | 80.3 | 22.4 | 80.4 | 22.1 | 81.0 | 21.5 | 0.966 |
| **Social functioning** | 84.6 | 23.1 | 84.2 | 22.0 | 88.1 | 20.1 | 0.248 |

**Supplementary Table 3: Mean quality of life domain scores per number of symptom categories reported**

|  | **Number of symptom categories*** | | | | | | | |  |
| --- | --- | --- | --- | --- | --- | --- | --- | --- | --- |
|  | **0** |  | **1-4** |  | **5-8** |  | **8-11** |  |  |
| **Group size n, (%)** | 12 (3) |  | 142 (37) |  | 193 (50) |  | 42 (11) |  |  |
|  | **mean** | **SD** | **mean** | **SD** | **mean** | **SD** | **mean** | **SD** | **p** |
| **Global health status** | 91.7 | 8.7 | 84.0 | 13.5 | 71.3 | 16.3 | 64.9 | 19.8 | <0.001 |
| **Physical functioning** | 96.7 | 5.3 | 89.9 | 10.7 | 79.2 | 18.0 | 74.5 | 16.0 | <0.001 |
| **Role functioning** | 97.2 | 9.6 | 91.0 | 15.4 | 73.4 | 24.5 | 66.3 | 27.4 | <0.001 |
| **Emotional functioning** | 97.9 | 5.2 | 92.3 | 12.1 | 77.8 | 20.7 | 67.0 | 23.5 | <0.001 |
| **Cognitive functioning** | 95.8 | 7.5 | 92.7 | 13.0 | 73.6 | 22.1 | 63.1 | 24.0 | <0.001 |
| **Social functioning** | 100.0 | 0.0 | 94.6 | 12.1 | 80.6 | 22.9 | 71.8 | 29.3 | <0.001 |
| * Categories of health symptoms as described in Table 2 (11 catagories in total). Health symptoms were categorized in ten categories based on organ system. | | | | | | | | | |

**Supplementary Table 4: frequent reported (by ≥40% of respondents) dyads of categories of health symptoms**

|  | **Symptom categories*** | | | | | | | | | | |
| --- | --- | --- | --- | --- | --- | --- | --- | --- | --- | --- | --- |
| % reported | **Fatigue** | **Cardiac** | **Respiratory** | **Gastro-intestinal** | **Renal and urinary** | **Central nervous system** | **Skin** | **Psycho-logical** | **Reproductive system** | **Breast** | **Musculo-skeletal** |
| **Fatigue** |  |  |  |  |  | 54 |  | 43 | 44 | 42 | 53 |
| **Cardiac** |  |  |  |  |  |  |  |  |  |  |  |
| **Respiratory** |  |  |  |  |  |  |  |  |  |  |  |
| **Gastro-intestinal** |  |  |  |  |  |  |  |  |  |  |  |
| **Renal and urinary** |  |  |  |  |  |  |  |  |  |  |  |
| **Central nervous system** |  |  |  |  |  |  |  | 46 | 46 | 42 | 55 |
| **Skin** |  |  |  |  |  |  |  |  |  |  |  |
| **Psycho-logical** |  |  |  |  |  |  |  |  | 40 |  | 45 |
| **Reproductive system** |  |  |  |  |  |  |  |  |  |  | 45 |
| **Breast** |  |  |  |  |  |  |  |  |  |  | 45 |
| **Musculo-skeletal** |  |  |  |  |  |  |  |  |  |  |  |
| * Categories of health symptoms as described in Table 2 (11 categories in total). Health symptoms were categorized in ten categories based on organ system. Empty cells represent dyads of health symptoms that were less frequently reported (<40%). | | | | | | | | | | | |

**Supplementary Table 5: multivariate linear regression for the effect of reported health problems on EORTC-QLQ-C30 quality of life domains, including the corrections for covariates**

|  | **Quality of life domain (β, CI) →** | **Global health status** | **Physical functioning** | **Role functioning** | **Emotional functioning** | **Cognitive functioning** | **Social functioning** |
| --- | --- | --- | --- | --- | --- | --- | --- |
| **Age at diagnosis** | **<50** | ref | ref | ref | ref | ref | ref |
|  | **50-59** |  | -0.8  ( -5.1 ; 3.6) |  |  | 3.0  (-3.0 ; 9.1) | 5.5  (-1.0 ; 12.0 |
|  | **60-69** |  | **-6.8***  (-11.0 ; -2.5) |  |  | **7.4***  (1.5 ; 13.4) | 6.6 (0.1 ; 13.0) |
|  | **70+** |  | **-13.5***  (-18.1 ; -8.9) |  |  | **7.8***  (1.4 ; 14.3) | 0.6 (-6.6 ; 7.8) |
| **Time since diagnosis** | **<2** | ref | ref | ref | ref | ref | ref |
|  | **2-4** |  |  |  |  |  |  |
|  | **>4** |  |  |  |  |  |  |
| **Highest completed level of education** | **Secondary education or lower** | ref | ref | ref | ref | ref | ref |
|  | **Medium vocational training (MBO)** |  |  |  |  |  | -2.0  (7.2 ; 3.2) |
|  | **High vocational training (HBO/ university)** |  |  |  |  |  | **-5.6***  (12.3 ; -0.8) |
| **Comorb** | **Yes** | **-5.5***  (-8.6 ; -2.4) | **-6.1***  (-9.0 ; -3.3) | **-8.5***  (12.9 ; -4.1) |  |  |  |
| **IBR** | **Yes** | **7.7***  (2.3-13.0) |  |  | **8.4***  (2.3 – 14.5) |  |  |
| **Health problems (in categories)** | **Fatigue/**  **endurance** | **-9.4** (-13.0 ; -5.8) | **-8.2**  (-11.2 ; -3.3) | **-14.1**  (-18.9 ; -9.3) |  |  | **-6.0**  (-10.2 ; -1.7) |
|  | **Cardiac** | **-4.4** (-8.3 ; -0.5) |  |  | **-5.5**  (-9.9 ; -1.0) | **-5.7**  (-10.5 ; -0.8) |  |
|  | **Respiratory** |  |  |  |  |  |  |
|  | **Gastrointestinal** | **-3.4** (-6.8 ; -0.1) | **-5.3**  (-8.2 - -2.4) | **-7.8**  (-12.3 ; -3.3) |  | **-4.3**  (-8.5 ; -0.1) |  |
|  | **Renal and urinary** |  |  |  |  | **-9.7**  (-16.5 ; -2.8) |  |
|  | **Central nervous system** | **-5.5** (-9.3 ; -1.7) |  |  | **-8.3**  (-12.5 ; -4.1) | **-16.2**  (-20.8 ; -11.6) | **-6.1**  (-11.2 ; -0.9) |
|  | **Skin** |  |  |  |  |  |  |
|  | **Psychological** |  |  |  | **-12.7**  (-12.7 ; -8.8) | **-5.2**  (-9.3 ; -0.9) | **-6.2**  (-10.8 ; -1.7) |
|  | **Reproductive system** |  |  |  |  |  |  |
|  | **Breast** |  |  |  |  |  |  |
|  | **Musculoskeletal** | **-4.2** (-8.0 ; -0.5) | **-7.6**  (-11.0; -4.3) | **-9.5**  (-14.7 ; -4.3) | **-5.0**  (-9.1 ; -0.8) |  | **-6.3**  (-11.4 ; -1.2) |
| CI: confidence interval; IBR: immediate breast reconstruction; comorb: comorbid diseases at time of questionnaire  Factors that were significant in univariate testing (p<0.10) were included in multivariate testing. Cells are empty when factors were not significant in univariate testing and thus not included in multivariate testing, or were excluded through backward selection in the multivariate analyses.  Reference categories for health problems were patients who not reported health problems in this category.  Corrected for age at diagnosis, presence of comorbid diseases, highest completed education at time of diagnosis, and breast reconstruction.  * significant in multivariate testing (level of significance in multivariate testing: 0.05).  ^1^ in years | | | | | | | |
